# Schizoaffective Disorder in an acute psychiatric unit: Profile of users and agreement with Operational Criteria (OPCRIT)

**DOI:** 10.4102/sajpsychiatry.v22i1.790

**Published:** 2016-05-06

**Authors:** Ryola Singh, Ugasvaree Subramaney

**Affiliations:** 1 Department of Psychiatry, School of Clinical Medicine, Faculty of Health Sciences, University of Witwatersrand, South Africa; 2 Sterkfontein Hospital, Krugersdorp, South Africa

## Abstract

**Background:**

Schizoaffective Disorder is a controversial and poorly understood diagnosis. Experts disagree on whether it is a discrete disorder; whether it is on a spectrum between Bipolar Disorder and Schizophrenia or whether it even exists. Lack of individual research attention given to this disorder, changing diagnostic criteria and hence poor diagnostic stability have all contributed to the dearth of knowledge surrounding Schizoaffective Disorder.

**Objectives:**

To describe the profile of mental health care users (MHCUs) diagnosed with Schizoaffective Disorder and determine the degree of agreement between the clinicians’ diagnosis and Operational Criteria (OPCRIT).

**Method:**

All MHCUs at Helen Joseph Hospital psychiatric unit with Schizoaffective Disorder between 01 January 2004 and 31 December 2010 were included. The demographic, clinical and treatment profiles as well as data required for OPCRIT were extracted from hospital records and discharge summaries.

**Results:**

Most MHCUs with Schizoaffective Disorder were female (68.89%), with a mean age of illness onset of 25 years (SD ± 7.11), had a family history of mood disorders (76.92%) and displayed impaired functioning. Majority (80%) were treated with at least one antipsychotic and one mood stabiliser. No agreement was found between the clinicians’ diagnosis and OPCRIT.

**Conclusion:**

While the profile of MHCUs with Schizoaffective Disorder in this study is similar to other studies, the lack of agreement between the clinicians’ and OPCRIT diagnoses calls for further research using larger population samples and a dimensional approach to diagnoses in order to improve understanding and management of Schizoaffective Disorder.

## Introduction and background

In 1933, Jacob Kasanin reported on nine cases that were diagnosed with Dementia Praecox, but who seemed quite atypical.^1^ They presented with sudden onset of ‘schizophrenic’ and affective symptoms following emotional and environmental stressors. The psychosis lasted a few weeks to a few months and was followed by recovery. Kasanin described them as having ‘acute schizoaffective psychoses’ and suggested that they were an intermediate group, with their outcome being better than Schizophrenia and worse than mood disorders.

Decades after ‘Schizoaffective Disorder’ was coined by Kasanin, it remains poorly understood. Some consider it to be in the middle of a continuum between Schizophrenia and Bipolar Disorder or a heterogeneous group of the two.^2^ Others believe it to be a psychotic mood disorder and not a discrete entity.^3^

Schizoaffective Disorder has moreover demonstrated poor diagnostic stability (36%) using DSM IV consensus diagnoses assessed by psychiatrists who were blind to previous research diagnoses.^4^ Furthermore, the inter-rater reliability for the individual items of the DSM-IV Schizoaffective Disorder criteria assessed by two psychiatrists using the Composite International Diagnostic Interview was found to be low (Cohen’s kappa of 0.22).^5^

A re-evaluation of 59 MHCUs’ hospital records of patients diagnosed with Schizoaffective Disorder using OPCRIT as well as agreement by two psychiatrists, found that only 6 users met the ICD-10 criteria and none met the DSM IV criteria for the disorder on a lifetime basis.^6^

The current concept of Schizoaffective Disorder is quite disparate from that originally illustrated by Kasanin.^1^ However, Kasanin has provided us with the reality that there is a subset of MHCUs that do not fit into the once popular, neat, dichotomous categories of having either a psychotic disorder or a mood disorder. For decades this subset of users has sparked controversy and hence, has been neglected in psychiatric research. Fortunately, interest in the Schizoaffective Disorder construct has been gaining momentum in recent times and attempts have been made to demystify the illness.^7^ However, we are not yet at the juncture where we can make firm conclusions or give clear guidelines based on the current available evidence.

Ensuring an accurate diagnosis of Schizoaffective Disorder is imperative and perhaps should be the starting point as this has implications for treatment planning and prognosis. It may also allow for more robust research in the future and hence a greater understanding of the disorder.

To date, no studies have examined the agreement of Schizoaffective Disorder between the clinician and OPCRIT in South Africa. The acute psychiatric unit at Helen Joseph Hospital is the point of entry for most MHCUs living in that catchment area and is representative of other acute psychiatric units in Johannesburg. This study sought to provide a local perspective and shed some clarity on the diagnosis and profile of MHCUs with Schizoaffective Disorder.

## Method

This study used the DSM IV-TR (text revision) definition of Schizoaffective Disorder as this is what was in use at the study site at the time.^8^

Data was retrieved from the hospital records of all MHCUs with either a discharge, differential or previous routine clinical diagnosis of Schizoaffective Disorder admitted to Helen Joseph Hospital between 01 January 2004 and 31 December 2010. The rationale for also including patients with a diagnosis of Schizoaffective Disorder both previously or as a differential diagnosis was to prevent poor diagnostic stability and poor reliability of retrospective data from excluding likely candidates. Also, if only a discharge diagnosis was considered, the sample size would have been even smaller (*N* = 28). The profile of these users was described and computer-generated DSM IV diagnoses were extracted from OPCRIT.

OPCRIT was developed in 1991 by McGuffin et al utilising a poly-diagnostic approach to psychiatric disorders.^9^ OPCRIT has shown good reliability (kappa 0.73)^10^ and excellent agreement with what some authors deemed the ‘gold standard’ consensus best-estimate lifetime diagnosis.^11^

OPCRIT for Windows version 4.0 is a 90-item checklist of demographic data, psychopathology, pre-morbid functioning, stressors prior to illness onset, personal history and family history. It has built-in algorithms designed to generate reliable diagnoses from case notes according to operational criteria of 12 major classification systems (e.g. DSM IV, ICD-10). It has not incorporated DSM IV TR criteria, but no differences exist between the DSM IV and DSM IV TR criteria for Schizoaffective Disorder. Symptoms are recorded for the time frame being studied (e.g. most recent episode, worst ever episode etc.). This study used ‘other specified episode’ which recorded the episode that had the most information in the users’ files. Considering Schizoaffective Disorder is a longitudinal illness, OPCRIT has additional questions to account for this such as: total duration of illness (including prodromal, residual and active phases of illness and course of the disorder (including single episode with good recovery, multiple episodes with good recovery between, multiple episodes with partial recovery between, continuous chronic illness and continuous chronic illness with deterioration).

OPCRIT guidelines state:

The package is specifically for the needs of the researcher and is intended to be used by clinicians or investigators trained in clinical research. It is not recommended for use by raters without previous experience in psychopathology and psychiatric diagnosis.

Also, if one is unfamiliar with OPCRIT, the 90 questions programme should be initially used, where data is entered item by item and an adjacent information button provides additional guidance. Therefore, previous experience in psychopathology and use of the guided questions made OPCRIT easy to use despite being unfamiliar with the tool prior to this study.

## Statistical analysis

The data was analysed using Statistica version 8.0. Descriptive analysis used frequencies and percentages for categorical variables, and means (standard deviation) or medians (range) where appropriate. Agreement between the clinicians’ diagnosis and OPCRIT was assessed using the kappa coefficient which measures the agreement between two raters and takes into consideration the agreement occurring by chance. It ranges from 0 (no agreement) to 1 (complete agreement).

### Ethical clearance

Ethics approval was obtained from the University of Witwatersrand’s Human Research and Ethics Committee.

## Results

**FIGURE 1:**
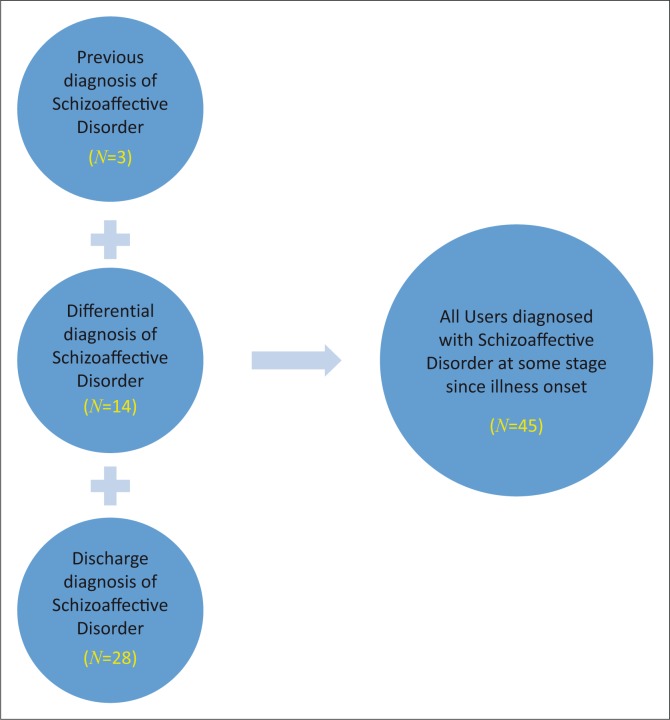
Sampling method.

Ninety users were identified, however, only 45 were included as the remaining files were untraceable. These 45 users included those with a diagnosis of Schizoaffective Disorder at discharge (*N* = 28), as a differential diagnosis (*N* = 14) or as noted in the past psychiatric history (*N* = 3) (Figure 1).

### Demographic Profile

Of the sample (*N* = 45), 68.89% (*N* = 31) were female (Table 1). The mean age was 44 years (SD ± 12.28), and majority (84.44%, *N* = 38) were single. Only 11.11% (*N* = 5) were employed despite 79.07% (*N* = 34) having 10 years or more of education. Data regarding disability grants were only available for 34 users of which 64.71% (*N* = 22) were receiving a disability grant.

**TABLE 1:** Demographic profile of mental health care Users (*N* = 45).

Variable		Number (%)
Age (mean ± SD) (years)		44 ± 12.28
Gender [*n* (%)]	Females	31 (68.89)
	Males	14 (31.11)
Marital Status [*n* (%)]	Single	38 (84.44)
	Married	7 (15.56)
	Level of Education [*n* (%)] < 10 years*	9 (20.93)
	≥ 10 years*	34 (79.07)
Unemployed [*n* (%)]		40 (80.89)
On a Disability Grant [*n* (%)]†		22 (64.71)

* Data absent in file for 2 users.

† Data absent in file for 11 users.

### Psychiatric History

The mean age of illness onset was 25 years (SD ± 7.11), (range 14–44 years) (Table 2). The mean number of days spent in hospital was 23 days (range 2–53 days). Many users (60%, *N* = 27) had 5 or more psychiatric admissions. A Substance Use Disorder was present in 24.44% (*N* = 11) of users but inadequate information precluded further analysis of this.

**TABLE 2:** Psychiatric History of Mental Health Care Users (*N* = 45).

Variable	Number (%)
Age of Onset of Mental Illness (mean ± SD) (years)*	25 ± 7.11
Number of Days in Hospital (mean ± SD)	23 ± 12.77
Number of Admissions [*n* (%)] < 5	18 (40)
≥ 5	27 (60)
Positive Family Psychiatric History [*n* (%)]	25 (55.56)
Presence of Substance Use Disorders [*n* (%)]	11 (24.44)

* Data absent in file for 2 users.

### Family Psychiatric History

A family history of mental illness was present in 55.56% (*N* = 25) of the users. Of those for which information was available (52% (*N* = 13), 76.92% (*N* = 10) had a family history of a mood disorder: 46.15% (*N* = 6) with Bipolar Disorder and 30.77% (*N* = 4) with Depression. A family history of Schizophrenia was present in 7.70% (*N* = 1) of users, while 15.38% (*N* = 2) had a family history of both Bipolar Disorder and Schizophrenia.

## Clinical Profile of Users

### Diagnostic Profile

**FIGURE 2:**
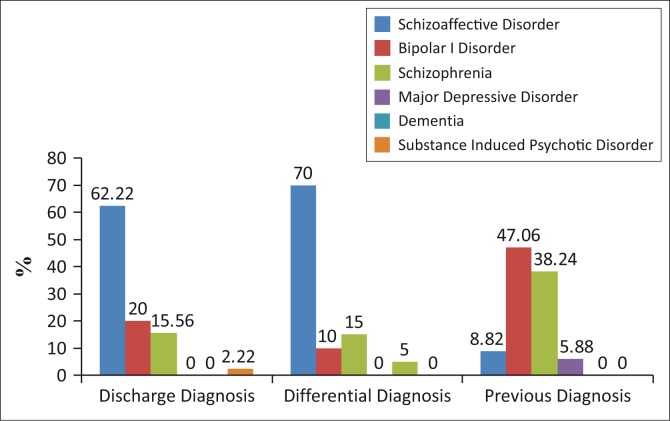
Diagnostic profile.

Discharge diagnoses were as follows: Schizoaffective disorder 62.22% (*N* = 28), Schizophrenia 20% (*N* = 9), Bipolar Disorder 15.56% (*N* = 7) and Substance-induced Psychotic Disorder 2.22% (*N* = 1) (Figure 2). The Schizoaffective Disorder subtype was specified for 75% (*N* = 21) of users, with 90.48% (*N* = 19) having the Bipolar and 9.52% (*N* = 2) having the Depressive subtype.

Differential diagnoses were given to 44% (*N* = 20) of the sample and were as follows: Schizoaffective Disorder (70% (*N* = 14), Bipolar I Disorder (15%, *N* = 3), Schizophrenia (10%, *N* = 2) and Dementia (5%, *N* = 1).

Diagnoses on previous admissions available for 76% (*N* = 34) of users follows: Schizophrenia 47.06% (*N* = 16), Bipolar I Disorder 38.24% (*N* = 13), Major Depressive Disorder 5.88% (*N* = 2) and Schizoaffective Disorder 8.82% (*N* = 3).

## Treatment Profile

### Number of Neuroleptics

Most MHCUs (77.78%, *N* = 35) were treated with 1 antipsychotic while 22.22% (*N* = 10) were treated with two (Table 3). Flupenthixol Decanoate (Fluanxol) or Zuclopenthixol Decanoate (Clopixol) were the second antipsychotics used in those that were treated with two antipsychotics. Majority (80%, *N* = 36) were on a combination of at least 1 mood stabiliser and 1 antipsychotic. No mood stabiliser was prescribed in 20% (*N* = 9) of users, while 73.33% (*N* = 33) received one mood stabiliser and 6.67% (*N* = 3) received two. Of those on two mood stabilisers (*N* = 3), two were treated with a combination of Sodium Valproate and Lithium and one with Carbamazepine and Lithium. An antidepressant was prescribed for 11.11% (*N* = 5) of users.

**TABLE 3:** Number of Neuroleptics (*N* = 45).

Variable	Number	Result (%)
Number of Antipsychotics [*n* (%)]	0	0 (0)
	1	35 (77.78)
	2	10 (22.22)
Number of Mood Stabilisers [*n* (%)]	0	9 (20)
	1	33 (73.33)
	2	3 (6.67)
Number of Antidepressants [*n* (%)]	0	40 (88.89)
	1	5 (11.11)
	2	0 (0)
Combination of at least 1 Antipsychotic and 1 Mood Stabiliser [*n* (%)]	-	36 (80)

### Antipsychotics

Risperidone was most commonly prescribed (40%, *N* = 18); followed by Haloperidol (33.33%, *N* = 15); Flupenthixol Decanoate depot (26.67%, *N* = 12); Zuclopenthixol Decanoate Depot (8.89%, *N* = 4); Clozapine (8.89%, *N* = 4); Trifluoperazine (2.22%, *N* = 1) and Risperidone Consta (2.2%, *N* = 1).

### Mood Stabilisers

Majority (60%, *N* = 27) of users received Sodium Valproate while the remainder received Carbamazepine (13.33%, *N* = 6), Lithium (11.11%, *N* = 5) or Lamotrigine (2.22%, *N* = 1).

### Antidepressants

Citalopram was prescribed for 8.89% (*N* = 4) of MHCUs and Mianserin for 2.22% (*N* = 1).

## Further Treatment

Upon discharge, 55.56% (*N* = 25) were referred to their local psychiatric clinic for follow up and 11.11% (*N* = 5) were referred to a psychiatric placement facility. The remaining MHCUs were transferred to other psychiatric hospitals for further management.

**FIGURE 3:**
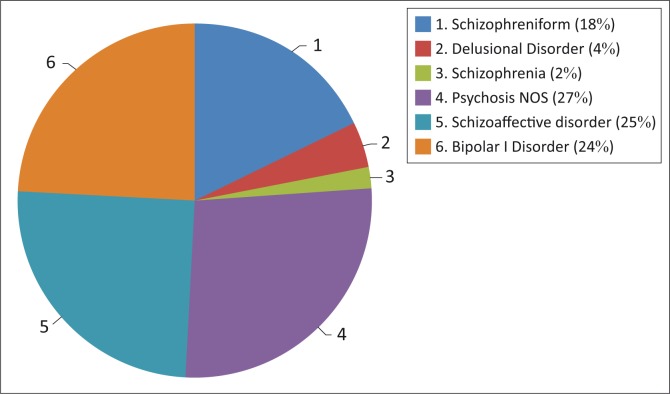
Operational Criteria (OPCRIT) diagnoses.

The most common diagnosis generated by OPCRIT was Psychosis Not Otherwise Specified (26.67%, *N* = 12) (Figure 3). Schizoaffective Disorder and Bipolar I Disorder were each diagnosed in 24.44% (*N* = 11) of users. The least common diagnoses generated were Schizophreniform Disorder (17.78%, *N* = 8), Delusional Disorder (4.44%, *N* = 2) and Schizophrenia (2.22%, *N* = 1).

Entering data into OPCRIT from hospital records was challenging due to lack of detailed information and may explain why Psychosis Not Otherwise Specified (NOS) was the most common diagnosis generated. Information on ‘relation between mood and psychotic symptoms’ an important criterion used to make the diagnosis of Schizoaffective Disorder, was absent for 27.27% (*N* = 12) of users.

### Agreement between Clinician and OPCRIT

The Kappa Coefficient was found to be (-0.0014), indicating no agreement between the clinicians’ diagnosis and OPCRIT.

## Discussion

### Main Findings

Epidemiological data on Schizoaffective Disorder is lacking and collating data is difficult due to poor diagnostic reliability, stability and changing diagnostic criteria.^7^ In this sample, females predominated, in keeping with other studies.^6,7^ The majority being single, unemployed and receiving a disability grant suggests that they experienced social and occupational dysfunction and may have a poorer outcome. This is consistent with the Vollmer-Larsen study^6^ but contrasts Kasanin’s original description.^1^ Unfortunately, the available literature has been inconsistent in predicting outcomes in these users.^12,13,14^

The broad range of illness onset (14–44 years) is noted elsewhere, with a third being similar to that of Schizophrenia and a third being similar to that of Bipolar Disorder, which again makes categorising Schizoaffective Disorder difficult.^7^

Despite the results suggesting a higher preponderance of mood disorders in the MHCUs’ families, substantial conclusions cannot be drawn as data was absent for 48% of this sub-sample. The fact that these users had both a family history of Schizophrenia and Bipolar Disorder suggests that Schizoaffective Disorder has a link to both mood and psychotic disorders. This is in line with newer evidence demonstrating abnormalities in the gene DISC1 (Disrupted in Schizophrenia 1) in Schizophrenia, Bipolar Disorder and Schizoaffective Disorder.^15^

This sample received various diagnoses at different admissions. Other studies have also documented this diagnostic instability, citing illness evolution, new information being presented and poor reliability of cross-sectional diagnoses as contributory factors.^4,6^ The DSM IV criteria has been criticised for not being specific enough regarding the proportion of the duration of the mood episodes to the total duration of the active and residual phases of the illness. The DSM-5 has addressed some of these concerns.^16^ However, what constitutes ‘majority’ of total duration of illness is not specified in DSM-5 and making the diagnosis still relies heavily on there being adequate historical information as well as clinical judgment, which requires a degree of experience.

A systematic review of randomised, double-blind, placebo-controlled trials of treatment specific for Schizoaffective Disorder as well as post-hoc analysis of similar trials for Schizophrenia and Bipolar Disorder noted that research is greatly lacking on specific treatment for Schizoaffective Disorder.^17^ They found the most robust evidence for the use of atypical antipsychotics and highlighted that despite ‘classical’ mood stabilisers (e.g. Lithium and anticonvulsants) being commonly prescribed in clinical practice, there is no good-quality evidence to support this.

A review of clinical practice data for the treatment of Schizoaffective Disorder revealed that 93% of users received an antipsychotic, 48% received a mood stabiliser and 42% received an antidepressant.^18^ The findings of this study are somewhat in keeping with these prescribing trends as all users received at least one antipsychotic and 80% received at least one mood stabiliser.

The finding that OPCRIT most commonly diagnosed Psychosis Not Otherwise Specified (26.67%) while Schizoaffective Disorder was diagnosed in 24.44% of users, is not surprising, given the ambiguities and complexities of diagnosing Schizoaffective Disorder. However, insufficient, retrospective data, with the absence of information regarding relationship between mood and psychotic symptoms for 27% of the sample, is also likely to have contributed to this finding. This information is integral to the diagnosis of Schizoaffective Disorder and is treated by the scoring program as 0 if absent. Certainly, supplementing the OPCRIT with a clinical interview and reaching a consensus best-estimate lifetime diagnosis would have been ideal.

Vollmer-Larsen et al also found no agreement between the clinicians’ diagnosis and OPCRIT.^6^ The advantage of their study is that they supplemented this with a consensus diagnosis by two psychiatrists who had reviewed the files. Being able to do this using retrospective records implies that the records contained adequate and relevant information, which was not possible to do in this study.

The lack of agreement between the clinicians’ and OPCRIT diagnoses in this study may seem trivial, but one should also consider the underlying implications of this finding. Schizoaffective Disorder is deemed to be rare, ambiguously defined, inadequately studied and therefore poorly understood. Hence, concurrence on the diagnosis of Schizoaffective Disorder cannot be expected at this point.

### Limitations

This retrospective study lends itself to many challenges which could have influenced the results including: the quality of the information in the files, histories and mental state examinations done by doctors with different degrees of experience and accuracy of assessments and the temporal relationship between mood and psychotic features often not being clearly documented. Also, information on possible confounding factors such as the impact of substance use disorders and pre-existing or co-morbid medical conditions were scant.

Another important limitation is the small sample size, which precludes any meaningful interpretations or conclusions being made from these results.

Of course, having no prior experience or training in the use of OPCRIT is also a valid limitation, but this is not a prerequisite for use of the program. Clinical experience and use of the guided 90-item questions are considered adequate to use OPCRIT.

## Recommendations

More research is urgently recommended in this area. South Africa has a rich heritage with differing cultures, ethnicities and socio-economic backgrounds. It may therefore prove useful to determine whether the epidemiology, illness pattern, treatment response and outcomes vary in this diverse population. The history of Schizoaffective Disorder, with its poor diagnostic stability and reliability is testament to the inadequacy of a categorical approach to psychopathology. Therefore, further studies on Schizoaffective Disorder alone, done longitudinally and using a more dimensional approach may garner greater understanding of Schizoaffective Disorder. The issue of poor inter-rater reliability and diagnostic stability can only be rectified with a clearer definition and objective measures such as genetic markers for the disorder – all of which require further research.

## Conclusion

The profile of these users is in many ways akin to descriptions in more contemporary international literature. These include a female preponderance of individuals with impaired functioning who have been given a wide variety of diagnoses over time. These findings are not generalisable however, and require replication using larger sample sizes and improved methodology in future studies. This research may not change our clinical practice, but highlights the need for greater vigilance with careful documentation of these users’ illness trajectory and treatment response.
